# Gcn4 impacts metabolic fluxes to promote yeast chronological lifespan

**DOI:** 10.1371/journal.pone.0292949

**Published:** 2023-10-13

**Authors:** Juan Facundo Gulias, Florencia Niesi, Martín Arán, Susana Correa-García, Mariana Bermúdez-Moretti

**Affiliations:** 1 Facultad de Ciencias Exactas y Naturales, Departamento Química Biológica, Universidad de Buenos Aires, Buenos Aires, Argentina–CONICET, Instituto de Química Biológica de la Facultad de Ciencias Exactas y Naturales (IQUIBICEN), Buenos Aires, Argentina; 2 Fundación Instituto Leloir e Instituto de Investigaciones Bioquímicas de Buenos Aires (IIBBA)—CONICET, Patricias Argentinas, Buenos Aires, Argentina; Western University, CANADA

## Abstract

Aging is characterized by a gradual decline in physiological integrity, which impairs functionality and increases susceptibility to mortality. Dietary restriction, mimicking nutrient scarcity without causing malnutrition, is an intervention known to decelerate the aging process. While various hypotheses have been proposed to elucidate how dietary restriction influences aging, the underlying mechanisms remain incompletely understood. This project aimed to investigate the role of the primary regulator of the general amino acid control (GAAC) pathway, the transcription factor Gcn4, in the aging process of *S*. *cerevisiae* cells. Under conditions of amino acid deprivation, which activate Gcn4, the deletion of *GCN4* led to a diverse array of physiological changes in the cells. Notably, the absence of Gcn4 resulted in heightened mitochondrial activity, likely contributing to the observed increase in reactive oxygen species (ROS) accumulation. Furthermore, these mutant *gcn4*Δ cells exhibited reduced ethanol production despite maintaining similar glucose consumption rates, suggesting a pivotal role for Gcn4 in regulating the Crabtree effect. Additionally, there was a marked reduction in trehalose, the storage carbohydrate, within the mutant cells compared to the wild-type strain. The intracellular content of free amino acids also exhibited disparities between the wild-type and *GCN4*-deficient strains. Taken together, our findings indicate that the absence of *GCN4* disrupts cellular homeostasis, triggering significant alterations in interconnected intracellular metabolic pathways. These disruptions have far-reaching metabolic consequences that ultimately culminate in a shortened lifespan.

## Introduction

In most living organisms, aging is characterized by a deterioration in physiological integrity and an increase in susceptibility to disease and death. Aging is the main risk factor for most diseases and conditions that limit longevity. Research in the field of aging has experienced unprecedented advances in recent years, mainly due to the discovery that the rate of aging is controlled, at least in part, by genetic pathways and biochemical processes conserved throughout evolution [1].

*Saccharomyces cerevisiae* has the peculiarity of being both a single cell and a complete organism, which has motivated its use as a tool for the study of aging [2]. Two models of aging that have similarities with different types of human cells are used [3,4]. Replicative lifespan (RLS) is based on counting the number of daughter cells that the same mother cell is capable of producing, or in other words, on quantifying the number of cell divisions that a single cell undergoes [2,4]. In chronological lifespan (CLS), cells that are not dividing but remain viable in stationary phase for a limited time are studied; that is, it is a measure of the maximum and average survival time of a population of cells that are not dividing [4,5].

Both aging models are strongly influenced by nutrient availability. Caloric restriction is one of the non-genetic interventions that extends longevity in a variety of species [6]. It is a type of dietary intervention in which calories are reduced while maintaining an adequate level of macronutrients [7]. In yeast, caloric restriction, which is achieved by decreasing the concentration of glucose in the culture medium, also increases longevity [8]. When the intervention being performed involves fasting other macronutrients such as protein, the intervention is called dietary restriction. Campos et al compared the effect of nitrogen source on CLS and found that the use of gamma-aminobutyric acid (restricted source) as the sole nitrogen source resulted in a longer lifespan than the use of glutamine (unrestricted source) [9]. In particular, reducing the availability of amino acids in the diet also increases longevity in yeast [8,10]. It has been demonstrated that reducing cellular translation through genetic and non-genetic interventions is a conserved mechanism of lifespan extension [11]. These interventions mediate their lifespan extending effect through the Ras-cAMP-protein kinase A (PKA) and target of rapamycin complex 1 (TORC1)-Sch9 pathways [3,12,13]. The Ras-PKA pathway acts in the sensing of nutrients (mainly glucose) and in the transduction of signals that promote growth, proliferation, and glycolysis. TORC1 regulates both cell volume and mass by participating in a variety of processes such as ribosome biogenesis and protein translation, nutrient sensing, mitochondrial metabolism and autophagy [14,15]. The treatment with the drug rapamycin, which downregulates the TORC1 pathway, also extends lifespan.

Stress is defined as a harmful condition, either environmental or physiological, that can affect the normal balance of an organism. There are several types of stress that contribute to aging such as osmotic stress, salt stress, thermal stress, endoplasmic reticulum (ER) stress, nutritional or metabolic stress, pH stress, and stress caused by toxic components such as toxic metals or metalloids and arsenical compounds [16]. Although exposure to different types of stress has clear harmful effects, in certain cases it has beneficial effects on longevity, depending on the duration and severity of the stress. The beneficial response to stress or toxin, which would cause a negative response at a higher exposure, is known as hormesis [17]. The intrinsic characteristics of stress, as well as its intensity and duration on the cell, are closely related to the impact and consequent response of the cell. This response, which in the first instance allows cell adaptation, can become detrimental to their fitness and survival. Several mechanisms that may contribute to transcriptional memory have been proposed in *S*. *cerevisiae* that could produce an effect both directly within the altered cell and be transmitted through multiple generations [16]. For example, exposure of yeast to low doses of DNA-damaging agents causing replicative stress has been shown to result in an increase in the number of quiescent cells in stationary phase [17].

The general amino acid control (GAAC) pathway, which regulates cellular amino acid homeostasis at a global level (reviewed in [18]) influences CLS. Activation of GAAC reduces CLS whereas suppression of GAAC extends CLS in minimal medium [19]. The main effector of this pathway is the transcription factor Gcn4. It has been proposed that the activation of Gcn4 translation, that depends on the activity of Gcn2, may be the consequence of a global response produced by the presence of different types of stress that produce hormesis in cells [20,21]. Gcn4 induces the expression of an important fraction of the yeast genome, which includes genes involved in the biosynthesis of amino acid or vitamin precursors, peroxisome component genes, mitochondrial carrier proteins, amino acid transporters, and proteins involved in autophagy. That is, although its main function is to elevate intracellular amino acid content, it also induces many other genes with no obvious connection to amino acid metabolism [22].

Because Gcn4 is an important regulator of amino acid and protein synthesis in response to different nutritional conditions, its involvement during the aging process has been the subject of study. Several reports that focused on elucidating the role of Gcn4 on longevity extension have arrived to diverse conclusions. Constitutively high-level expression of *GCN4* reduces chronological longevity, whereas suppression of GAAC activity extends longevity [19]. In contrast, Gcn4 is required to promote longevity in long-lived strains that are deficient for 60S ribosomal subunit, and also in long-lived cells subjected to caloric restriction or deficient in Tor1 or Sch9 that emulate this type of restriction [23]. Furthermore, Gcn4 promotes longevity by decreasing overall protein translation and the expression of genes involved in translation, and Gcn4 translation is upregulated in long-lived ribosomal protein deletion strains [11]. Also, it has been demonstrated that Gcn4 overexpression is sufficient to extend replicative lifespan in the absence of reduced global protein synthesis in an autophagy-dependent manner [24].

The participation of Gcn4 in the induction or repression of genes in response to different types of stress relates this transcription factor not only to longevity but also to autophagy. Gcn4 overexpression manages to promote replicative survival without inducing Gcn2 activity or inhibiting TORC1 activity but in an autophagy-dependent manner [24]. On the other hand, the stress generated in the endoplasmic reticulum due to the accumulation of misfolded proteins inside it, activates the UPR response (Unfolded Protein Response) whose functionality is necessary to prolong the chronological longevity of cells [25] and both Gcn2 and Gcn4 are required for the induction of most UPR target genes [26]. There is a crosstalk between GAAC and TORC1 pathways in *S*. *cerevisiae* [27]. Gcn2 activation occurs as a consequence of both the increase in the amount of tRNA released and the release of the inhibitory effect exerted by TORC1. The pioneering work of Valenzuela et al. [28] showed that rapamycin has the capability to induce *GCN4* translation. Staschke et al. analyzed the transcriptome during amino acid starvation and rapamycin treatment to investigate the integration of GAAC and TOR pathways [29]. Using prototrophic cells grown with ammonium as the nitrogen source, these authors discovered that GAAC functions as a major effector of the TOR pathway. They observed that Gcn4 induces a similar number of genes during rapamycin treatment compared to Gln3, a well-known target of TORC1. However, they observed also that a significant number of genes were regulated by Gcn4 in response to amino acid starvation induced by 3-aminotriazole (3-AT) but not in response to rapamycin treatment.

While its main role is to enhance intracellular amino acid levels, it concurrently activates numerous other genes that do not appear to be directly linked to amino acid metabolism. Given this broad influence of Gcn4, we embarked on a comprehensive study to investigate the potential impact of GCN4 deficiency on overall metabolism. Our objective was to explore whether the effects of Gcn4 deficiency extend beyond amino acid metabolism and stress responses, potentially providing insights into its influence on cellular lifespan. Our central hypothesis posits that the metabolic processes predominantly occurring during the logarithmic growth phase exert a significant influence on the determination of longevity. To investigate this hypothesis, we conducted a comprehensive comparison of proteomes, glucose consumption rates, ethanol production, respiratory activity, and the accumulation of free amino acids in wild-type and *gcn4*Δ strains during the exponential growth phase. Additionally, we assessed the accumulation of reactive oxygen species (ROS) and reserve carbohydrates during the stationary phase, which result from those metabolic processes. These measurements were performed during the stationary phase as it is when the consequential accumulation of ROS and reserve carbohydrates occurs as a consequence of that metabolic activity.The yeast growth condition chosen for this work was minimal medium with proline as the sole nitrogen source, in order to minimize the activity of TORC1. In addition, the growth medium was devoid of amino acids to work in conditions in which Gcn4 was active. Prototrophic strains were used in order to avoid the overall amino acid homeostasis perturbations that take place in auxotrophic strains [30]. Our results show that in the absence of amino acids, Gcn4 promotes chronological lifespan extension by altering the whole carbon flux in the cells.

## Materials and methods

### Strains

The *S*. *cerevisiae* strains 23344c (*Matα ura3*) [31], TOY01 (*Matα ura3 tor1*Δ::*KanMX4)* [32] and SBCY03 (*Matα ura3 gcn4*Δ::*KanMX4*) [33], isogenic to the wild-type Σ1278b, were used in this work.

Cells were grown in minimal medium containing 0.17% Difco yeast nitrogen base (YNB without amino acids and ammonium sulfate), 2% glucose as carbon source, 10 mM proline as nitrogen source and 0.002% uracil. When indicated, the medium was supplemented with the commercial mix of amino acids (-Ura Drop-out BD, 77 mg/100 ml of medium).

### Chronological life span determination

For chronological lifespan (CLS) assays, cells from fresh overnight cultures were inoculated in 20 ml of fresh medium in 125 ml-erlenmeyers with cotton covers to an optical density (OD) at 600 nm of 0.05. Cells were incubated at 30°C with shaking (200 rpm). After 72 hours of growth, when cells have reached the stationary phase and have stopped budding, cells were collected by centrifugation, resuspended in 20 ml of sterile water and kept at 30°C with shaking (200 rpm) throughout the entire experiment. This point was considered as 100% of survival (time 0). At the indicated times, aliquots were taken out from the suspensions to assess CLS by the colony forming units (CFU) spots method [34]. Briefly, from a 50-fold cellular dilution of each culture, five 10-fold dilutions (1:10) were serially prepared. 3 μl of each dilution were spotted in duplicate on YPD (1% yeast extract, 2% peptone, 2% glucose and 2% agar) plates and incubated for 2 days at 30°C. Colonies were counted in those spots with 6 to 30 colonies. The results were expressed as % Survival and are the means ± SEM of the total replicate samples coming from at least three independent experiments.

### Fluorescence microscopy

Cells were harvested by centrifugation and washed twice with fresh medium before incubation for 40 min at 30°C with 10 μM 2’,7’dichlorodihydrofluorescein diacetate (H_2_DCFDA) or 100 nM Mitotracker Red CMXROS. 10 μl of cell suspension were then immobilized onto a plate in the presence 1 mg/ml concanavaline and subjected to bright field and fluorescence microscopy. Cells were analyzed at 30°C on a Zeiss LSM980 microscope equipped with a 63X (NA 1.4) and appropriate filter sets for acquisition of DCF and MitoTracker Red fluorescence.

### Flow cytometry analysis

A total of 10^7^ cells were harvested by centrifugation and washed with fresh medium. For ROS analysis cells were resuspended in 1 ml fresh medium and treated in dark with 0.01 mM H_2_DCFDA for 40 min at 30°C. Then, cells were analyzed in a Flow Cytometer (Accuri C6 Plus Becton Dickinson, laser 488 nm/640nm). To distinguish living cells from dead ones propidium iodide (PI, SIGMA) was used. Washed cells were resuspended in 1 ml of PI staining solution (20 μg/ml). All cell suspensions were sonicated for 10 seconds before analysis. At least 5,000 events were counted. Negative controls with non-dyed cells were used as process checkup.

### Mass spectrometry analysis

Protein extraction was carried out as already described [35]. Briefly, total proteins were prepared by lysing yeast cells in 1.85 N NaOH-7.5% β-mercaptoethanol on ice for 10 min, followed by precipitation with trichloroacetic acid (TCA) at a final concentration of 8%. The TCA pellets were resuspended in sodium dodecyl sulfate (SDS) loading buffer. Extracts were subjected to SDS-PAGE. Protein digestion and Mass Spectrometry analysis were performed at the Proteomics Core Facility CEQUIBIEM, University of Buenos Aires/CONICET as follows: SDS-PAGE gel excised protein bands were reduced, alkylated in-gel digested with Trypsin. The recovered peptides were analyzed by HPLC-coupled mass spectrometry (Orbitrap QExactive technology coupled to nano-HPLC-ThermoScientific). The ionization of the samples was carried out using Electrospray. Mass spectra were analyzed with the Proteome Discoverer software, comparing against the *S*. *cerevisiae* proteome. In addition, the abundance of each protein was quantified based on the area calculation for each one. The normalized areas were statistically compared with Perseus software version: 1.5.8.5. [36]. A protein was considered to be differentially expressed if it presented a relative change equal to or greater than two in its expression level, with a *p* value less than 0.05 with the Student’s T-test.

### Determination of amino acids content

23344c and SBCY03 strains were cultured in minimal medium at 22°C until the OD600 reached 1. The cells were then centrifuged at 3500 x g for 10 min at 4°C. Cell pellets were washed twice with PBS buffer, resuspended in 2 ml ice-cold 80% methanol, disrupted by sonication and centrifuged at 4°C for 10 min at 15000 x g. Supernatants were collected and dried in a Savant SpeedVac (Thermo Scientific). Dried samples were solubilized in 0.5 ml sodium phosphate buffer (100 mM dissolved in D_2_O, pH = 7.4), supplemented with 2, 2-dimethyl-2-silapentane-5-sulfonate-d_6_ (DSS, final concentration 0.33 mM) as chemical shift reference. All NMR experiments were performed at 298 K on a Bruker Avance III spectrometer operating at a proton frequency of 600.2 MHz. ^1^H-NMR 1D spectra were acquired using a standard Bruker 1D NOESY pulse program with pre-saturation during relaxation delay and mixing time, and spoil gradients (noesygppr1d). The NMR data were zero-filled, Fourier transformed, phase-corrected using NMRPipe and converted to a Matlab-compatible format for further processing and analysis. All spectra were referenced to DSS (^1^H δ = 0 ppm) and submitted to water peak elimination, baseline correction and normalization. The assignment was achieved using the freely available electronic databases HMDB and BMRB, and subsequently confirmed by 2D spectra including heteronuclear single quantum coherence (HSQC) and total correlation spectroscopy (TOCSY). 2D ^1^H-^1^H TOCSY spectra were collected with N1 = 512 and N2 = 2048 complex data points. The spectral widths for the indirect and the direct dimensions were 9615.4 and 9604.9 Hz, respectively. The number of scans per t1 increment was set to 36. The transmitter frequency offset was 4.7 ppm in both ^1^H dimensions. 2D ^13^C-^1^H HSQC spectra were collected with N1 = 512 and N2 = 2048 complex data points. The spectral widths for the indirect and direct dimensions were 24906.9 and 12019.2 Hz, respectively. The number of scans per t1 increment was set to 256. The transmitter frequency offset was 70 ppm in the ^13^C dimension and 4.7 ppm in the ^1^H dimension.

The concentrations of the assigned metabolites were estimated using DSS as an internal reference standard and the statistical significance was assessed by Student´s t-test, taking p<0.05 as significant.

### Biochemical assays

For trehalose determinations, cells (50 mg wet weight) were collected and frozen in liquid nitrogen. After thaw on ice, samples were resuspended in 0.5 ml 0.25 M Na_2_CO_3_, incubated at 95°C for 20 minutes and centrifuged at maximal speed. 5 μl 1 N acetic acid and 5 μl buffer T (300 mM sodium acetate, 30 mM CaCl_2_ buffer pH 5.5) and 20 μl trehalase (aprox. 10 units) were added to 10 μl of the supernatant. Then, the reaction mix was incubated at 40°C for 2 hours, centrifuged and glucose was determined in the supernatant. The Wiener laboratory kit was used and the manufacturer´s guidelines were followed to quantify glucose.

Glycogen accumulation was detected by the brown colour produced by staining with iodine. Cells were collected and resuspended in a solution of 0.2% iodine/0.4% potassium iodide, incubated 3 min and then spotted onto YPD plates. The darker the color, the higher the amount of glycogen that was intracellularly accumulated [37].

Ethanol quantification was performed by a coupled reaction of 2 purified enzymes: *Pichia pastoris* alcohol oxidase (AOX) and radish peroxidase (HRP). Ethanol is a substrate for the AOX enzyme, which in the presence of O_2_ generates formaldehyde and H_2_O_2_. Hydrogen peroxide is a substrate for the HRP enzyme which, together with a colorless substrate, ABTS (2,2’-azino-bis(3-ethylbenzothiazoline-6-sulfonic acid)), generates a colored complex quantifiable by spectrometry [38]. To determine the standard curve, 30 μl of known concentrations of ethanol (0 mM, 2 mM, 4 mM, 8 mM and 10 mM) were plated in duplicate in a 96-well plate. On the other hand, serial dilutions were made to the medium of the supernatants obtained from the samples to be analyzed and 30 μl of each dilution were placed in duplicate in the 96-well plate. Then, 120 μl of the reaction mixture (5 mg/ml ABTS, 1.2 U/ml HRP and 0.3 U/ml AOX in 20 mM phosphate buffer pH 7) were placed in each well and the plate was incubated in the dark for 35 minutes at 30°C. To stop the reaction, 100 μl of 1% SDS were added and final product was measured by spectrometry at 412 nm.

## Results

**Fig 1:**
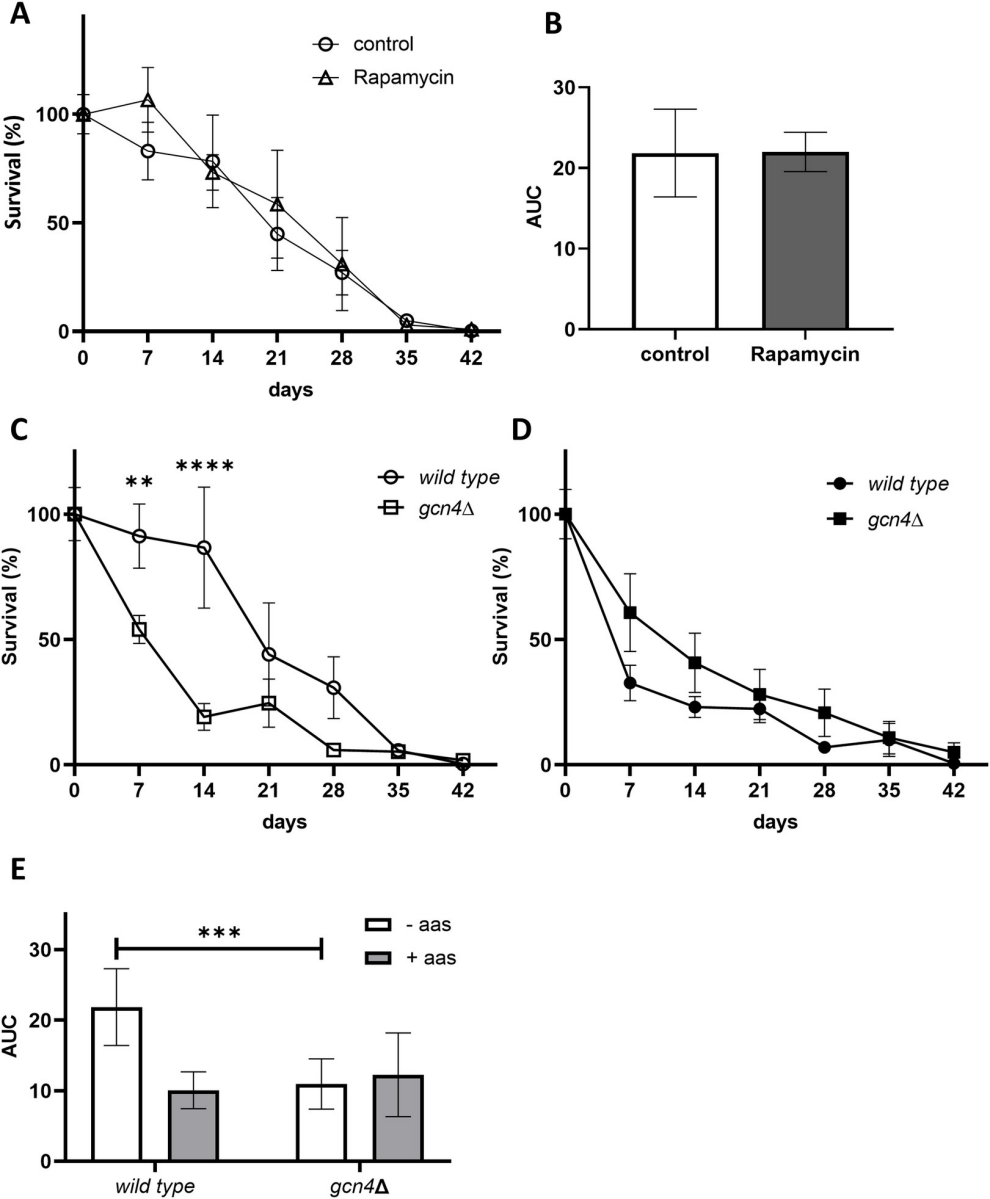
In the CLS model Gcn4 promotes longevity independently on TORC1. Chronological survival of wild-type and *gcn4*Δ strains was determined by the colony forming units (CFU) spots method. A: Wild-type cells grown without amino acids treated or not with rapamycin. B: Areas under the survival curves in A. C: Wild-type and *gcn4*Δ strains grown in the absence of amino acids. D: Wild-type and *gcn4*Δ strains grown in the presence of amino acids. E: Areas under the survival curves in C and D. The two-way ANOVA test (**: p<0.01; ***: p<0.001; ****: p<0.0001) was performed.

To conduct the CLS assays, we employed yeast cells that had reached the stationary phase in minimal media with proline as the sole nitrogen source. In a prior study, we demonstrated that under these growth conditions, the deletion of TOR1 did not impact longevity, as evidenced by the similar survival curves observed between wild-type and *tor1*Δ strains [39]. However, it is worth noting that TORC1 can be constituted not only by Tor1 but also by Tor2. As a preliminary step, we examined the influence of TORC1 on lifespan in wild-type cells cultivated in proline-based media. There was no effect of rapamycin on CLS (Figs 1A, 1B and S1). This result confirms that in our experimental conditions TORC1 was not active, as it is known that its activity has a strong effect on longevity. It should be noted that when we carried out a similar experiment but with wild-type cells grown with ammonium, the anti-aging effect of rapamycin was evident (S2 Fig). Then, we evaluated the effect of Gcn4 on CLS. Survival at the stationary phase significantly decreased in cells deficient in Gcn4 (Figs 1C and S1) and on day 28 of the CLS assay, we could no longer detect live *gcn4*Δ cells while viable wild-type cells remained. Moreover, the value of the area under the survival curves of the mutant strain was less than half that of the wild-type strain (Fig 1E). So, our results were similar to those already published that showed that Gcn4 promotes longevity in the CLS model [23] and demonstrated that the effect of Gcn4 on longevity was independent on TORC1 activity. It is worth noting that CLS of *gcn4*Δ and wild-type cells grown in the presence of a mixture of amino acids were indistinguishable (Figs 1D and S1) and that dietary restriction did not affect CLS in the mutant (Fig 1E). Since GAAC is induced by starvation of amino acids, it was expected that the effect of the lack of Gcn4 on longevity would be detected in the absence of the same.

**Fig 2:**
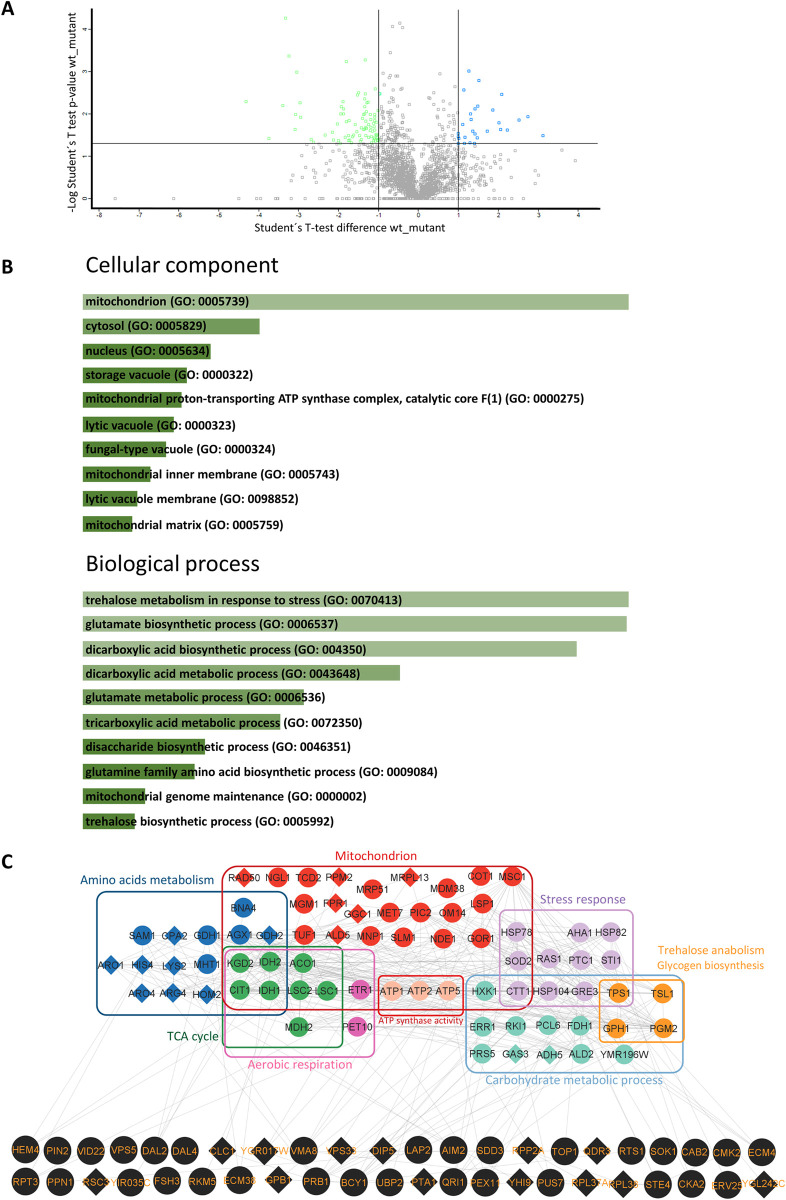
Proteomics analysis of *gcn4*Δ and wild-type yeast cells. A: The graph shows the result of the analysis obtained from the statistical comparison (T-test) for each protein of the samples from the wild-type and *gcn4*∆ strains grown until exponential phase (18 hours). The over-represented proteins in the mutant, that fell above the cutoff (a relative change wild type versus *gcn4*Δ of at least 2, with a value of p<0.05) are in quadrant 1, and those under-represented are in quadrant 2. B: Gene Ontology terms in which the differentially expressed proteins between wild-type cells and *gcn4*∆ cells are grouped. The bar graphs show the 10 most enriched terms from the list of differential proteins in each case. The length of the bars indicate how significant are the terms obtained from the analysis of the YeastEnrichr web platform (https://maayanlab.cloud/YeastEnrichr/). C: Classification of all proteins differentially expressed in the *gcn4*∆ mutant. Diamond-shaped proteins are those under-represented in the mutant, while the circular-shaped proteins are those over-represented. The proteins in black color correspond to those that could not be easily grouped with the rest. The protein interaction network was obtained using the STRING platform (https://string-db.org/) and modified using Cytoscape software.

Under nutrient-limiting conditions or other types of stress, Gcn4 induces the expression of an important fraction of the yeast genome, which includes genes that increase intracellular amino acid content but also induces many other genes with no obvious connection to amino acid metabolism. To study the mechanisms by which *GCN4* deficiency decreases longevity, we carried out a comparative study of wild-type and *gcn4*Δ proteomes using mass spectrometry with Orbitrap technology. We found 116 differentially expressed proteins between both strains (Fig 2A), 29 of them being under-represented in the *gcn4*∆ strain, and the other 87 being over-represented. Using the Regulatory Associations tool of the YEASTRACT platform [40], we found that some kind of regulation by Gcn4 had already been described for 114 of these 116 proteins. To our knowledge, there was no previous evidence of Gcn4 regulation of the gamma-glutamyl-transpeptidase Ecm38 or the mitochondrial endonuclease Ngl1 proteins, both of which were found to be over-represented in the mutant in our analysis. Gene Ontology (GO) analysis of the genes of the differentially expressed proteins (Fig 2B) and the interaction network between them (Fig 2C) showed an enrichment in several processes. Between them, amino acids metabolism was the most expected one.

To assess the impact resulting from the alteration in the relative quantities of proteins involved in amino acid metabolism, and acknowledging the critical role of amino acid balance in the regulation of yeast lifespan [41], we determined the intracellular amino acid content in both wild-type and *gcn4*∆ strains. To do this, we performed 1D NMR (Nuclear Magnetic Resonance) spectroscopy tests. The results show that there were significant differences between the concentrations of various but not all amino acids (Table 1). Both alanine and ornithine concentrations were found increased in *gcn4*∆ cells, whereas arginine, aspartate, glutamate, glycine, histidine, lysine and proline were found in lower concentrations in mutant cells. Lysine and arginine concentrations were the ones that suffered the greatest changes, since they decreased more than three times due to the absence of Gcn4. In this assay we were also able to quantify succinate and found that the concentration of this metabolite significantly decreased in the mutant. While Gcn4 is a known activator of amino acid biosynthetic genes, the overall content of free amino acids does not appear to significantly differ between the wild-type and *gcn4*∆ strains. However, despite the absence of a clear indication of reduced amino acid availability in the cytosol that promotes longevity, we cannot rule out the possibility that some of the observed changes may indeed impact CLS.

**Table 1:** Intracellular succinate and amino acids content in wild-type and *gcn4*Δ strains.

	wild type (mM)	*gcn4*∆ (mM)	*gcn4*∆/wild type	-log10(p)
Alanine ^a^	1.121	1.602	1.429	1.83
Ornitine ^a^	1.359	2.238	1.648	4.95
Succinic acid ^b^	0.255	0.081	0.316	2.69
Arginine ^b^	1.568	0.343	0.219	6.00
Asparagine ^b^	0.504	0.283	0.561	1.76
Glutamic acid ^b^	4.996	2.541	0.509	1.81
Glycine ^b^	0.409	0.225	0.549	1.55
Histidine ^b^	0.109	0.06	0.557	3.34
Lysine ^b^	1.517	0.444	0.293	4.13
Proline ^b^	6.524	1.558	0.239	2.50
Aspartic acid	0.043	0.061	1.410	
Glutamine	2.229	2.462	1.104	
Isoleucine	0.118	0.097	0.823	
Leucine	0.104	0.096	0.92	
Methionine	0.046	0.041	0.887	
Phenylalanine	0.052	0.051	0.986	
Serine	0.366	0.289	0.792	
Threonine	0.238	0.215	0.903	
Tyrosine	0.070	0.052	0.749	
Valine	0.186	0.165	0.887	

Only for significant differences -log10 (p) is shown.

^a^ increased levels in the mutant.

^b^ decreased levels in the mutant.

Gene Ontology (GO) analysis (Fig 2B) and the interaction network (Fig 2C) also showed an enrichment in mitochondrial proteins and suggested that processes such as aerobic respiration and carbohydrates metabolism, besides amino acids metabolism, might be altered in *gcn4*Δ cells.

**Fig 3:**
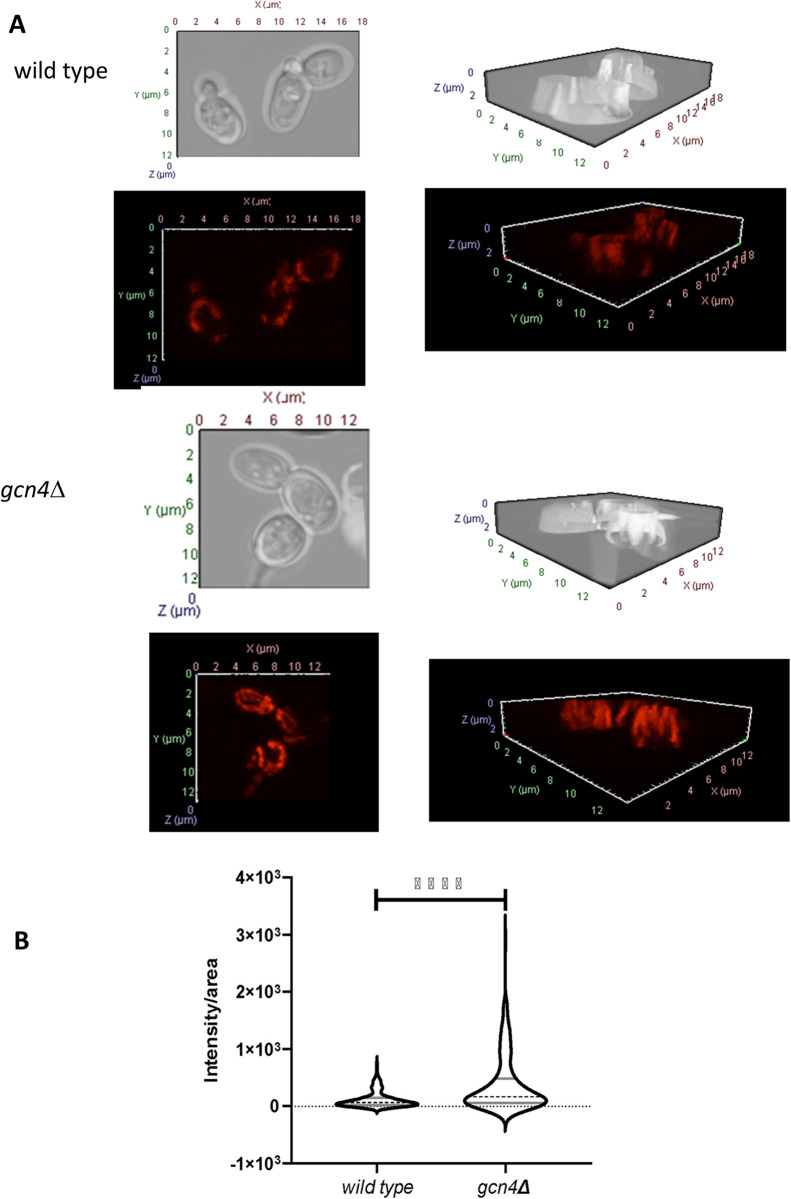
Mitochondrial activity is enhanced in *gcn4*Δ cells. A: Representative images taken under fluorescence confocal microscope of 3 wild type and *gcn4*∆ cells grown without amino acids until exponential phase (18 hours) and treated with 100 nM MitoTracker CMXRos are shown for exemplification. B: Violin diagrams show the distribution of the population density of wild-type and *gcn4*∆ cells. The dotted black line marking the mean value of each plot; the gray lines indicate the upper and lower quartiles. Significant differences (p<0.0001) between both strains were found using the two-way ANOVA test.

In view of the above, we decided to evaluate mitochondrial activity by fluorescence confocal microscopy in both wild-type and *gcn4*∆ cells grown in amino acids-restrictive conditions. To do this, these organelles were marked with the MitoTracker Red CMXRos reagent. This cationic and lipophilic fluorescent marker is concentrated within the mitochondria due to its negative mitochondrial membrane potential [42]. Thus, the more negatively charged the mitochondrial matrix, the greater the accumulation of the marker. *gcn4*∆ cells were marked in a greater proportion than wild-type cells and, in addition, the highest fluorescence intensities were observed in the mutant strain (Fig 3). These results suggested that mitochondrial respiratory activity under restrictive-conditions is increased in Gcn4-deficient cells. In cells grown with amino acids, *gcn4*∆ also showed more respiratory activity than wild-type (data not shown); however, this activity was significantly lower in this condition than in the restrictive one. Furthermore, differences in mitochondrial activity between the strains were also evident in cells during the stationary phase of growth (S3 Fig). At this stage of the growth curve, wild-type cells exhibited a greater mitochondrial membrane potential compared to the *gcn4*∆ mutants. However, what is particularly intriguing is the observation of mitochondrial morphology in both strains. While the majority of wild-type cells displayed a dynamic branched and tubular mitochondrial network, we observed the presence of small rounded vesicles in the *gcn4*∆ strain. This phenomenon, characterized by mitochondrial fragmentation, is known to be associated with age-dependent changes in cells [43,44]. In other words, the presence of vesicular mitochondria in *gcn4*∆ cells suggests that they were, on average, older than their wild-type counterparts. Importantly, this correlation aligns with the shorter lifespan of the mutant.

**Fig 4:**
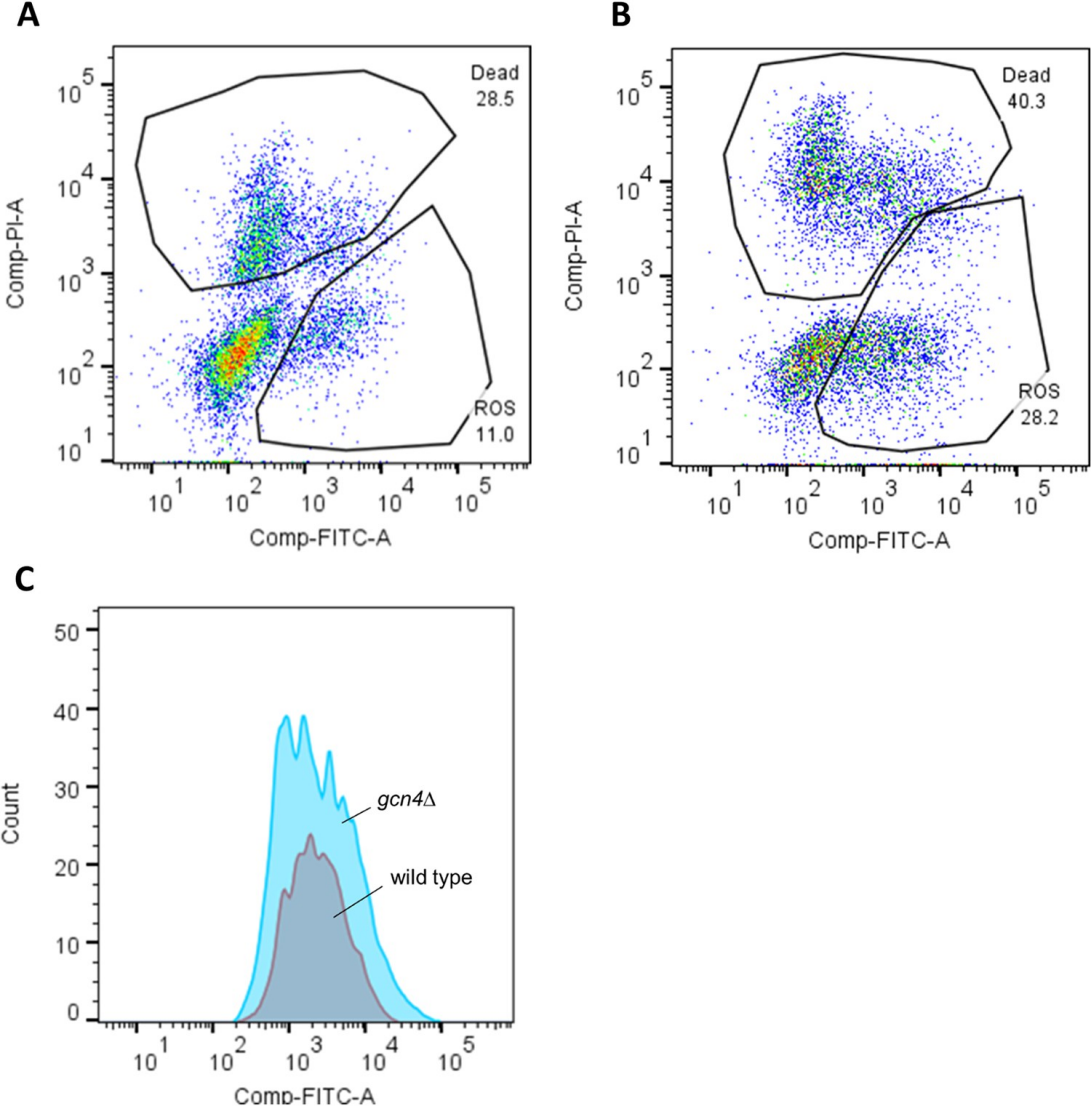
ROS accumulation is enhanced in stationary *gcn4*Δ cells. Representative graphs showing dot plots from flow cytometry analysis in wild-type (A) and *gcn4*∆ (B) cells grown without amino acids. Cells of interest were gated on SSC and FSC plot to remove any debris. ROS cells (positive for staining with H_2_DCFDA) and dead cells (positive for staining with propidium iodide) were defined by comparing with untreated cells. The x axis is a log scale of the intensity of H_2_DCFDA fluorescence and the y axis is a log scale of the intensity of propidium iodide fluorescence. The percentage of cells positive for each stain are indicated. In all cases, more than 10,000 cells were analyzed. C: Histogram comparing FITC signal of both strains.

Since mitochondria are the major intracellular source of reactive oxygen species (ROS), and it is extensively known that ROS damage cellular components and accelerate aging, we investigated *gcn4*∆ and wild-type cells by flow cytometry upon incubation with the ROS-specific probe H_2_DCFDA. Cells at exponential phase of growth were hardly stained (data not shown). So, we analysed ROS accumulation in cells at stationary phase (72 hours). The percentage of DCF-positive cells was 11.0% for wild-type cells and 28.2% for *gcn4*Δ cells indicating a strongly increased ROS accumulation in mutant cells (Fig 4). It is worth noting that cells grown in the presence of amino acids accumulated much less ROS (S4 Fig). By fluorescence microscopy, a much higher number of fluorescent *gcn4*Δ cells was imaged in comparison with wild-type cells (S4 Fig).

**Fig 5:**
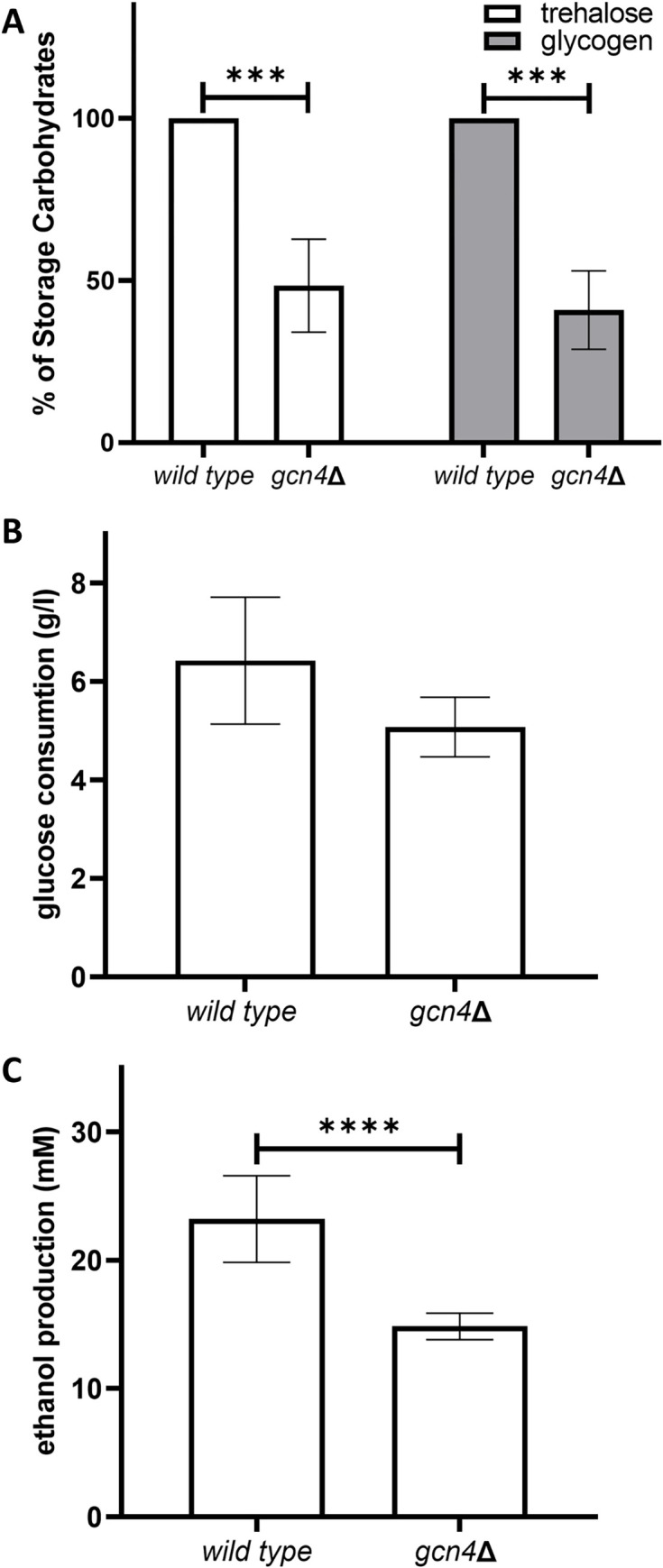
*gcn4*∆ cells accumulate less storage carbohydrates and produce less ethanol. Cells were grown in minimal medium without amino acids. A) Relative percentage of glycogen and trehalose contents in *gcn4*∆ cells with respect to wild-type cells at stationary phase (72 hours). B: Glucose concentration was determined in the culture medium of wild-type and *gcn4*∆ cells at exponential growth phase (18 hours). Glucose consumption was calculated as the difference between the initial glucose concentration and the concentration in each tested condition. Values indicate the average of at least 3 independent experiments and the deviation indicates the standard error of the mean (SEM). The two-way ANOVA test was performed. C: Ethanol concentration (mM) was determined in the culture medium of wild-type and *gcn4*∆ cells at exponential growth phase (18 hours). Values indicate the average of at least 3 independent experiments and the deviation indicates the standard error of the mean (SEM). The two-way ANOVA test (****: p<0.0001) was performed.

In addition mitochondrial activity and ROS accumulation, another characteristic of quiescent cells that contributes to longevity is the accumulation of storage carbohydrates. Cao et al. have previously demonstrated the importance of these storage carbohydrates, including trehalose and glycogen, for yeast cells’ survival during the stationary phase [45]. They found that efficient accumulation of these carbohydrates during the transition phases significantly impacts long-term survival. Given these insights and considering the differential expression of proteins involved in trehalose and glycogen metabolism in Gcn4-deficient cells (Fig 2B and 2C), we decided to assess the storage carbohydrate content in wild-type and *gcn4*∆ cells. We were unable to detect glycogen or trehalose in cells at exponential growth phase (18 hours) (data not shown). As it is known that both carbohydrates begin to be synthesized during the diauxic phase of growth and are consumed during the stationary phase when cells are deprived of nutrients [46], we performed the assays using cells at stationary phase (72 hours). We found a decrease of 50–60% in glycogen and trehalose contents in *gcn4*∆ cells with respect to wild-type cells (Fig 5A). This combined diminished accumulation of storage carbohydrates is probably implicated in the shortening of CLS in the mutant.

Also, several enzymes that participate in the tricarboxylic acid cycle were found over-represented in *gcn4*∆ cells. To further analyse the effect of Gcn4 in the carbon flux, we determined both glucose consumption and ethanol production. Although no significant differences in glucose concentration were detected in the culture medium of wild-type and *gcn4*∆ cells (Fig 5B), the mutant produced significantly less ethanol than the wild type (Fig 5C).

It must be noted that when cells were grown in the presence of amino acids, the accumulation of glycogen, glucose consumption and ethanol production were similar in wild-type and *gcn4*∆ cells (S5 Fig). Also, the difference in trehalose accumulation found under this growth condition was smaller to that observed under the more restrictive situation.

Altogether our results demonstrate that, under conditions in which TORC1 is inactive, Gcn4 has an important role in determining the metabolic state of cells, not only at the level of amino acids availability but also at the whole metabolism and the carbon flux, leading to an increase longevity.

## Discussion

In this work, we analyzed the role of Gcn4 on the chronological lifespan of prototrophic yeast cells under conditions in which TORC1 is down-regulated. Our findings clearly demonstrate that *GCN4* deficiency shortens the lifespan under these conditions. The lack of Gcn4 under amino acids fasting conditions significantly changed the expression level of 116 proteins. Among them, the mitochondrial proteins Atp1, Atp2 and Atp5, which are part of the mitochondrial ATP synthase, were over-represented. ATP synthase is an important complex that makes up about a quarter of mitochondrial membrane proteins and plays a central role in the production of energy used to drive cellular processes involving the generation of more than 90% of ATP in eukaryotic cells [47].

Some of the proteins with the highest relative abundance in *gcn4*∆ cells were enzymes that participate in the tricarboxylic acid (TCA) cycle. These differentially expressed enzymes sequentially catalyze steps in the cycle from the formation of citrate from oxaloacetate and acetyl-CoA (Cit1) to the oxidation of succinyl-CoA to form succinate (Aco1, Idh1/Idh2, Kgd2, Lsc1/Lsc2). These enzymes catalyze one-way reactions cycle suggesting that the synthesis of succinate could be increased in the mutant. However, the amount of succinate accumulated in *gcn4*∆ cells not only is not greater, but it is significantly smaller than that of wild-type cells. Whether this is because its synthesis is not increased, because its degradation is greater, or both, remains to be known.

The mitochondrial superoxide dismutase Sod2 and cytoplasmic catalase Ctt1 enzymes, both involved in redox processes, were also over-represented in the *gcn4*∆ strain. Sod2 catalyzes the formation of hydrogen peroxide from superoxide ion while Ctt1 reduces hydrogen peroxide to water. It is well known that during aerobic growth *SOD2* and *CTT1* genes are transcriptionally induced as a defense mechanism against oxidative damage [48]. We also detected a higher relative abundance of proteins involved in NAD/NADH metabolism in the mutant. Both the protein Bna4, which catalyzes the third step of the synthesis of NAD from tryptophan, and the enzymes Prs5, Rki1 and Gnd2, involved in the synthesis of phosphoribosyl pyrophosphate (PRPP), which is a necessary precursor for the *de novo* synthesis of NAD^+^ [49], were over-represented. The enzymes Nde1, Gut2 and Ndi1 are also over-represented in *gcn4*∆ cells. The cytosolic NADH produced in aerobiosis is transported and oxidized to NAD+ in mitochondria and this process is carried out mainly by two different systems that are coupled to the respiratory chain and have the enzymes Nde1/2 and Gut2 as effectors, respectively [50,51]. On the other hand, the NADH produced within the mitochondria is oxidized by the action of the enzyme Ndi1, which, like Nde1/2, is coupled to the respiratory chain and catalyzes the transfer of electrons from NADH to ubiquinone without the translocation of protons through the inner mitochondrial membrane [52]. The possible increase in NADH production together with the fact that the already mentioned subunits of ATP synthase, as well as the enzymes Sod2 and Ctt1, were over-represented, well correlate with the increased respiratory activity we detected occurring in the *gcn4*∆ mutant. Sod2 and Ctt1 overexpression in the mutant might be a consequence of ROS accumulation in these cells.

Other pathways involved in carbon flux appear to be modified in *gcn4*∆ cells. The enzymes Gph1 and Pgm2, both over-represented in *gcn4*∆ cells, participate in glycogen catabolism. Gph1 catalyzes the formation of glucose 1-phosphate from the glycogen pool [46], and Pgm2 interconverts glucose 1-phosphate and glucose 6-phosphate [53]. Therefore, the decrease in glycogen accumulation during the stationary phase in *gcn4*∆ cells is in agreement with the increased expression of these enzymes. In contrast, despite the overexpression of two subunits of the complex involved in trehalose synthesis, Tsl1 and Tps1, less trehalose accumulated in the mutant, which could contribute to its shorter chronological longevity. During starvation, both reserve carbohydrates can serve as long-term carbon reserves during starvation. But trehalose also acts as a molecular chaperone [54] and protects cells from stress [55,56]. Furthermore, Cao et al demonstrated that trehalose accumulation plays a fundamental role in the extension of CLS in *S*. *cerevisiae* [45].

As expected, the absence of Gcn4 modified the free pool of some, but not all, amino acids. From these, only alanine and ornithine concentrations were found increased. Hepowit and collaborators demonstrated that pharmacological lowering amino acid pools enhances lifespan of budding yeast [30]. Under our experimental conditions proline is the only available nitrogen source. Therefore, its nitrogen is used by the cells to synthesize all nitrogenous compounds. Cells can internalize proline both through the general amino acid permease Gap1 and through the proline-specific transporter Put4. Once inside the cell, the mitochondrial enzymes Put1 and Put2 degrade proline, generating glutamate [57]. We found here that *gcn4*∆ cells accumulate less proline and glutamate than wild type cells. The increase in the amount of ornithine in *gcn4*∆ cells could explain, at least in part, why glutamate decreases despite the relative increase in Gdh1 enzyme expression. Ornithine, which is synthesized from glutamate, and the compound carbamoyl phosphate are used for the synthesis of citrulline, a precursor of arginine. The decrease in the *gcn4*∆ mutant of the expression of the enzyme Cpa2, which catalyzes the synthesis of carbamoyl phosphate from glutamine, could cause a lower amount of carbamoyl phosphate, decrease the synthesis of citrulline and, as a consequence, cause the ornithine accumulation observed. In addition, the diminished abundance of the enzyme Arg4, which catalyzes the last step of arginine biosynthesis, could also contribute to a lower arginine synthesis, which we see reflected in the lower relative amount of arginine in *gcn4*∆ cells. On the other hand, the decrease in the expression of the enzymes Gdh2, which synthesizes α-ketoglutarate from glutamate, and Lys2, which catalyzes the fifth step of lysine biosynthesis, correlates with the decreased amount of lysine in *gcn4*∆. Furthermore, lysine biosynthesis consumes glutamate in both the fourth and sixth steps. There is evidence that, regardless of the conditions studied, Gcn4 promotes the synthesis of amino acids, in particular, lysine and arginine [58]. Moreover, these authors proposed that a decrease in arginine and lysine is one of the mechanisms that cause the decrease in protein translation in *gcn4*∆ mutant cells, since the molecular machinery involved in this process is enriched in these amino acids.

Although it is known that during aging, mitochondrial function is gradually lost, at the point in growth curves in which we measured this activity, *gcn4*∆ cells presented a greater mitochondrial activity than the wild-type strain. Mitochondrial ROS production capacity is determined by membrane potential [59]. This may probably be the reason why the mutant accumulates more ROS. ROS react with macromolecules causing serious damage to nucleic acids, lipids, and proteins [60], which justifies that the cells that accumulate ROS are less long-lived. In addition to mitochondrial activity, we found other metabolic changes generated by the absence of Gcn4. We found that although glucose consumption is similar in wild-type cells and in Gcn4-deficient mutants, ethanol production is lower in mutants. In other words, part of the consumed glucose, which in wild-type cells ends up in ethanol and trehalose, in the *gcn4*∆ strain has a different destination. In ethanolic fermentation, pyruvate from glycolysis is decarboxylated to acetaldehyde, which is mostly reduced to ethanol although a small portion is oxidized to acetate. It remains to be answered if acetate formation is increased in *gcn4*∆ cells, but if so, this would also be contributing to cell death since acetic acid is a stress and death inducing agent [61]. However, it should be mentioned that the enzymes Ald5 and Adh5, which catalyze the synthesis of acetate and ethanol from acetaldehyde, respectively, were found under-represented in the mutant proteome. Previously, Martínez et al constructed a causal model of the molecular events that trigger the aerobic production of ethanol in the presence of repressing levels of glucose (Crabtree effect) in *S*. *cerevisiae*. This model revealed a central role of three transcription factors involved in activating the amino acid metabolism, being Gcn4 one of them [62].

Amino acid fasting can be considered a type of hormesis that, as a consequence, generates a global decrease in protein synthesis. The latter generates the activation of Gcn4 translation [18]. In this work we demonstrate that the effect of Gcn4 goes beyond the deficiency of amino acids and the response to stress, since it also affects the flow of carbon metabolism, which could be the main mechanism by which Gcn4 regulates longevity. In this way, in conditions in which TORC1 is not active, Gcn4 would be promoting longevity, in a similar way, at least in part, to that of the anti-aging drug, rapamycin. As mentioned above, the effect of rapamycin on longevity is generated by many of the cellular responses that are triggered by nutrient starvation, such as inhibition of protein synthesis, down-regulation of amino acid permeases, protein degradation, autophagy, cell cycle arrest and higher ROS level [63,64].

Taking all our results together, we can conclude that the absence of Gcn4 initially leads to an imbalance in amino acid metabolism, primarily causing a decrease in intracellular concentrations of several amino acids. To compensate for this and maintain adequate translation levels, *gcn4*Δ cells should replenish intracellular amino acids by activating biosynthetic pathways that provide the carbon skeletons for de novo synthesis of amino acids, utilizing nitrogen donor molecules such as glutamine, aspartate, glutamic acid, or ammonium. One of the main pathways providing these carbon skeletons is the TCA cycle, which is clearly activated in *gcn4*Δ cells. The activation of the TCA cycle likely leads to a high reducing content that, in turn, activates the mitochondrial respiratory chain, resulting in elevated ATP levels.

Despite succinate being formed during the TCA cycle, its concentration diminishes in *gcn4*Δ cells. This decrease might be occurring due to succinate interacting with Complex II of the respiratory pathway to produce more cellular energy [65]. A high mitochondrial respiratory activity is associated with a high production of ROS [66]. However, ROS production was not observed in gcn4 cells during the exponential phase. This could be a consequence of the activity of enzymes that protect cells from oxidative damage, such as Sod2 and Ctt1, which are over-expressed in the mutant strain. The fact that ROS was only detected at the stationary phase could be the result of a diminished activity of such enzymes, probably due to the oxidative damage these enzymes themselves could suffer or to a slow translation rate that normally occurs at this growth phase.

In summary, the short longevity observed in cells deficient in Gcn4 is likely a consequence of their high respiratory activity, driven by the cells in an attempt to maintain adequate translation levels to survive. We propose that Gcn4 activity induced by amino acid-restrictive conditions generates changes in the general metabolism of cells, affecting not only amino acid metabolism but also regulating pathways that determine the cellular energy state. All these changes combined may lead to the extension of chronological longevity.

## Supporting information

S1 Fig Chronological lifespan. CLS was assayed for wild-type and *gcn4*Δ yeast strains grown in minimal medium supplemented or not with amino acids for 72 hours and then, transferred to water. At the indicated times, 3 μl of 10-fold serial dilutions were spotted onto rich media (YPD) agar plates and grown for 48 hours at 30°C followed by image capture. These are representative images from three or more experiments.
(TIF)

S2 Fig Chronological lifespan. A: CLS was assayed for wild-type yeast cells grown in minimal medium with ammonium as the sole nitrogen source treated or not with rapamycin and then, transferred to water. At the indicated times, 3 μl of 10-fold serial dilutions were spotted onto rich media (YPD) agar plates and grown for 48 hours at 30°C followed by image capture. B: Survival curves corresponding to results shown in A.
(TIF)

S3 Fig Mitochondrial activity. A: Representative images taken under fluorescence confocal microscope of wild type and *gcn4*∆ cells at stationary phase (72 hours) and treated with 100 nM MitoTracker CMXRos are shown for exemplification. B: Violin diagrams show the distribution of the population density of wild-type and *gcn4*∆ cells. The dotted black line marking the mean value of each plot; the gray lines indicate the upper and lower quartiles. Significant differences (p<0.0001) between both strains were found using the two-way ANOVA test.
(TIF)

S4 Fig ROS accumulation in stationary *gcn4*Δ cells. Representative images of cells grown in minimal medium without amino acids analyzed on a Zeiss LSM980 microscope equipped with a 63X (NA 1.4) and appropriate filter sets for acquisition of DCF (A). Representative graphs showing dot plots from flow cytometry analysis in wild-type (B) and *gcn4*∆ (C) cells grown in the presence of amino acids. Cells of interest were gated on SSC and FSC plot to remove any debris. ROS cells (positive for staining with H_2_DCFDA) and dead cells (positive for staining with propidium iodide) were defined by comparing with untreated cells. The x axis is a log scale of the intensity of H_2_DCFDA fluorescence and the y axis is a log scale of the intensity of propidium iodide fluorescence. The percentage of cells positive for each stain are indicated. In all cases, more than 10,000 cells were analyzed. D: Histogram comparing FITC signal of both strains.
(TIF)

S5 Fig Accumulation of storage carbohydrates and production of ethanol. Cells were grown in minimal medium with amino acids A: Relative percentage of glycogen and trehalose contents in *gcn4*∆ cells with respect to wild-type cells at stationary phase (72 hours) grown in the presence of amino acids. B: Glucose concentration was determined in the culture medium supplemented with amino acids of wild-type and *gcn4*∆ cells at exponential growth phase (18 hours). Glucose consumption was calculated as the difference between the initial glucose concentration and the concentration in each tested condition. Values indicate the average of at least 3 independent experiments and the deviation indicates the standard error of the mean (SEM). The two-way ANOVA test was performed. C: Ethanol concentration (mM) was determined in the culture medium supplemented with amino acids of wild-type and *gcn4*∆ cells at exponential growth phase (18 hours). Values indicate the average of at least 3 independent experiments and the deviation indicates the standard error of the mean (SEM). The two-way ANOVA test (**: p<0.01) was performed. D: Representative image of one of the glycogen determinations. Samples were subjected to Lugol’s reagent. Duplicate seeding of wild-type and *gcn4*∆ cells treated with undiluted Lugol’s reagent, as well as half (1:2) and quarter (1:4) dilutions, are shown.
(TIF)
