# Supplementary figures and images for: Gcn4 impacts metabolic fluxes to promote yeast chronological lifespan
Source: PLoS One. 2023 Oct 13;18(10):e0292949. doi: 10.1371/journal.pone.0292949 (PMC10575530; doi:10.1371/journal.pone.0292949)

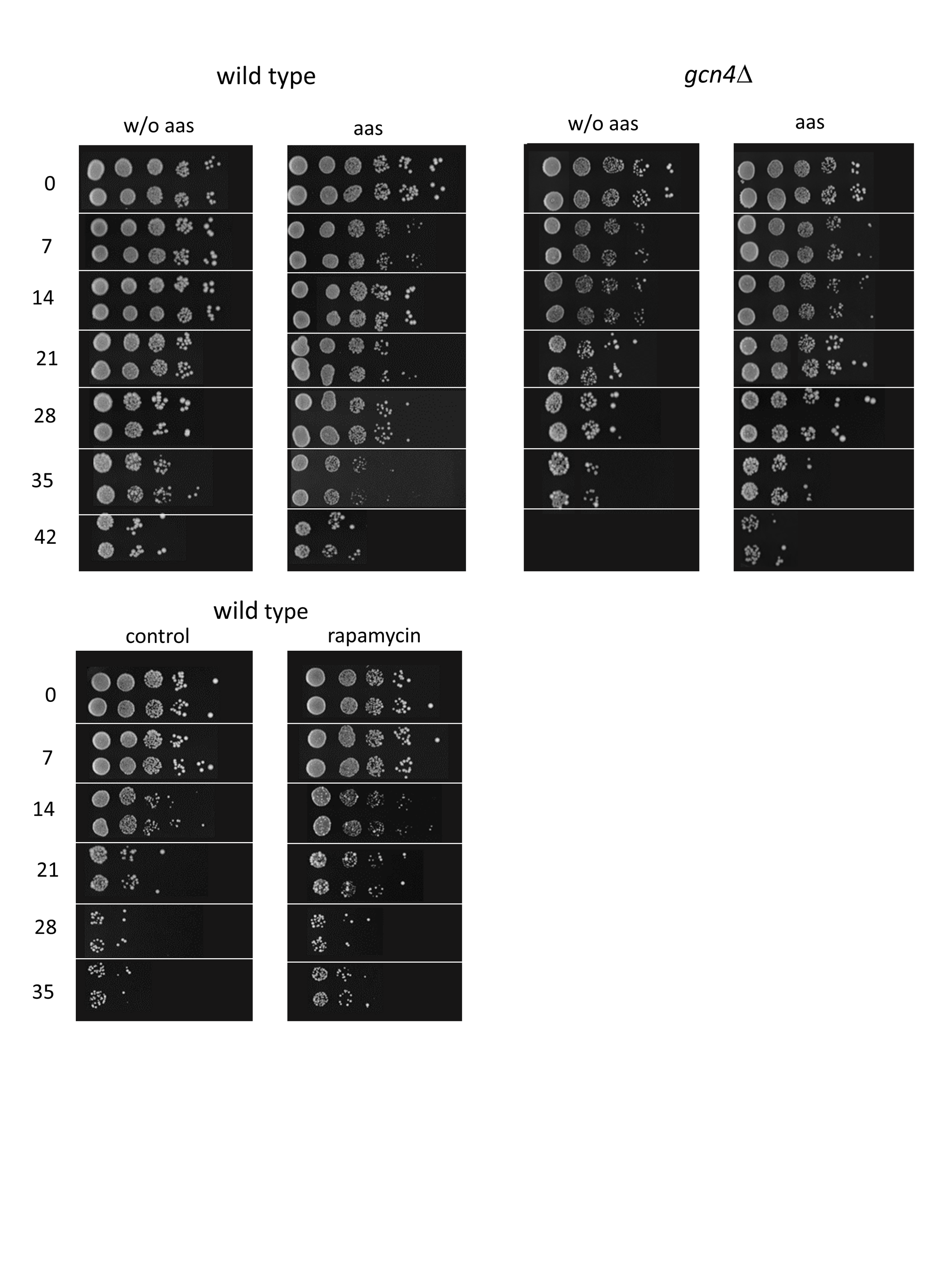

Supplement: S1 Fig — CLS was assayed for wild-type and gcn4Δ yeast strains grown in minimal medium supplemented or not with amino acids for 72 hours and then, transferred to water. At the indicated times, 3 μl of 10-fold serial dilutions were spotted onto rich media (YPD) agar plates and grown for 48 hours at 30°C followed by image capture. These are representative images from three or more experiments. (TIF) [file pone.0292949.s001.tif]

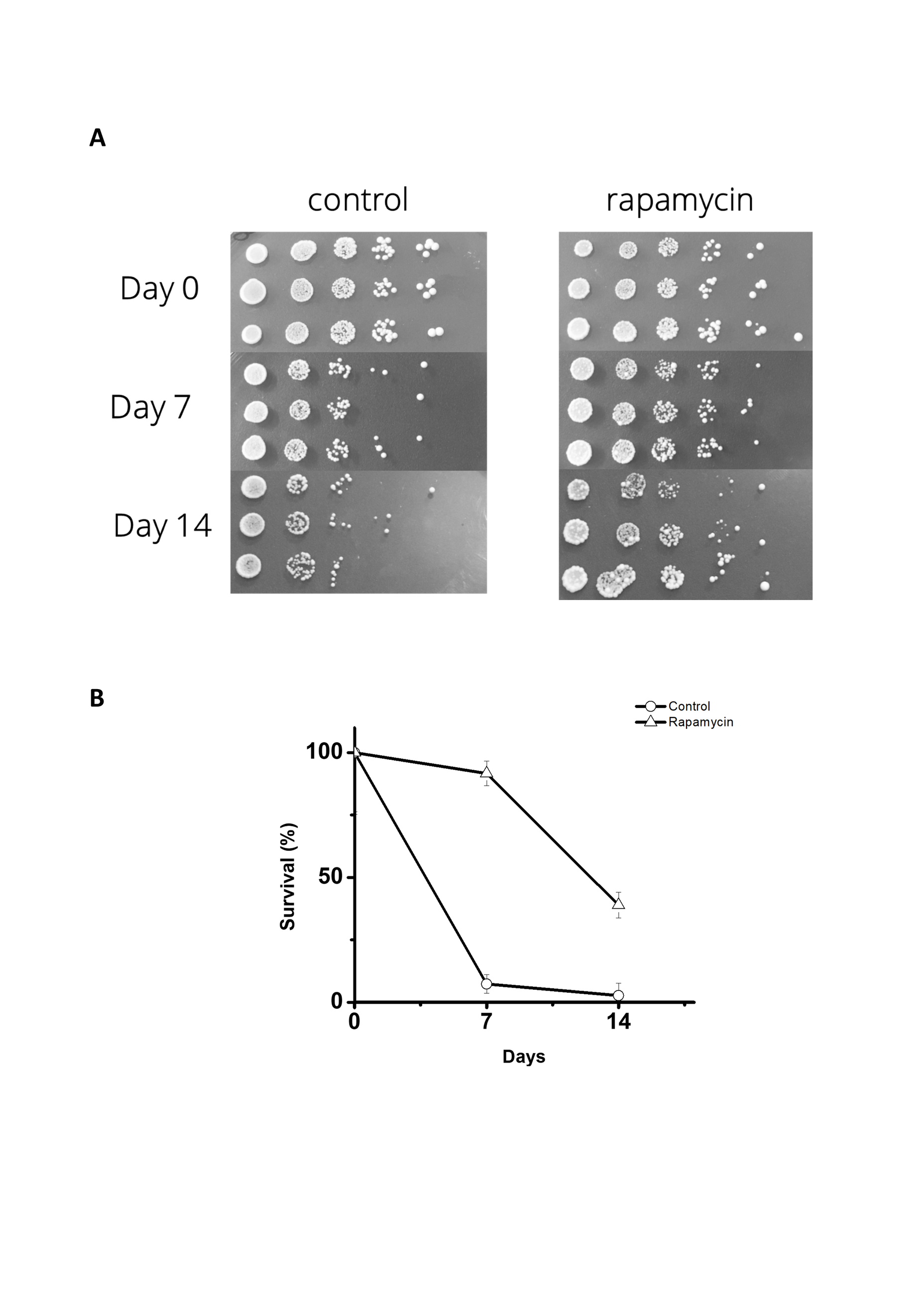

Supplement: S2 Fig — A: CLS was assayed for wild-type yeast cells grown in minimal medium with ammonium as the sole nitrogen source treated or not with rapamycin and then, transferred to water. At the indicated times, 3 μl of 10-fold serial dilutions were spotted onto rich media (YPD) agar plates and grown for 48 hours at 30°C followed by image capture. B: Survival curves corresponding to results shown in A. (TIF) [file pone.0292949.s002.tif]

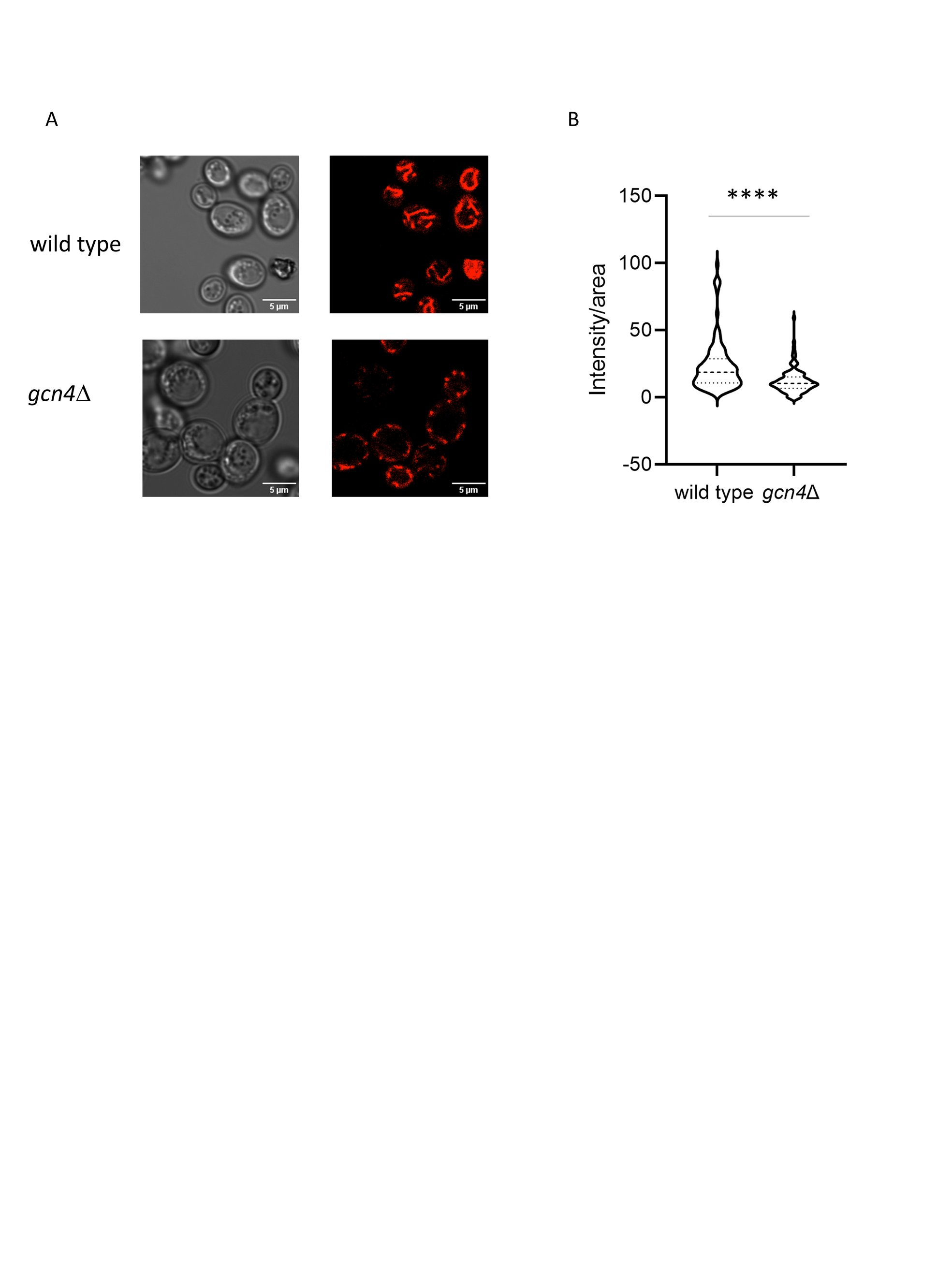

Supplement: S3 Fig — A: Representative images taken under fluorescence confocal microscope of wild type and gcn4∆ cells at stationary phase (72 hours) and treated with 100 nM MitoTracker CMXRos are shown for exemplification. B: Violin diagrams show the distribution of the population density of wild-type and gcn4∆ cells. The dotted black line marking the mean value of each plot; the gray lines indicate the upper and lower quartiles. Significant differences (p<0.0001) between both strains were found using the two-way ANOVA test. (TIF) [file pone.0292949.s003.tif]

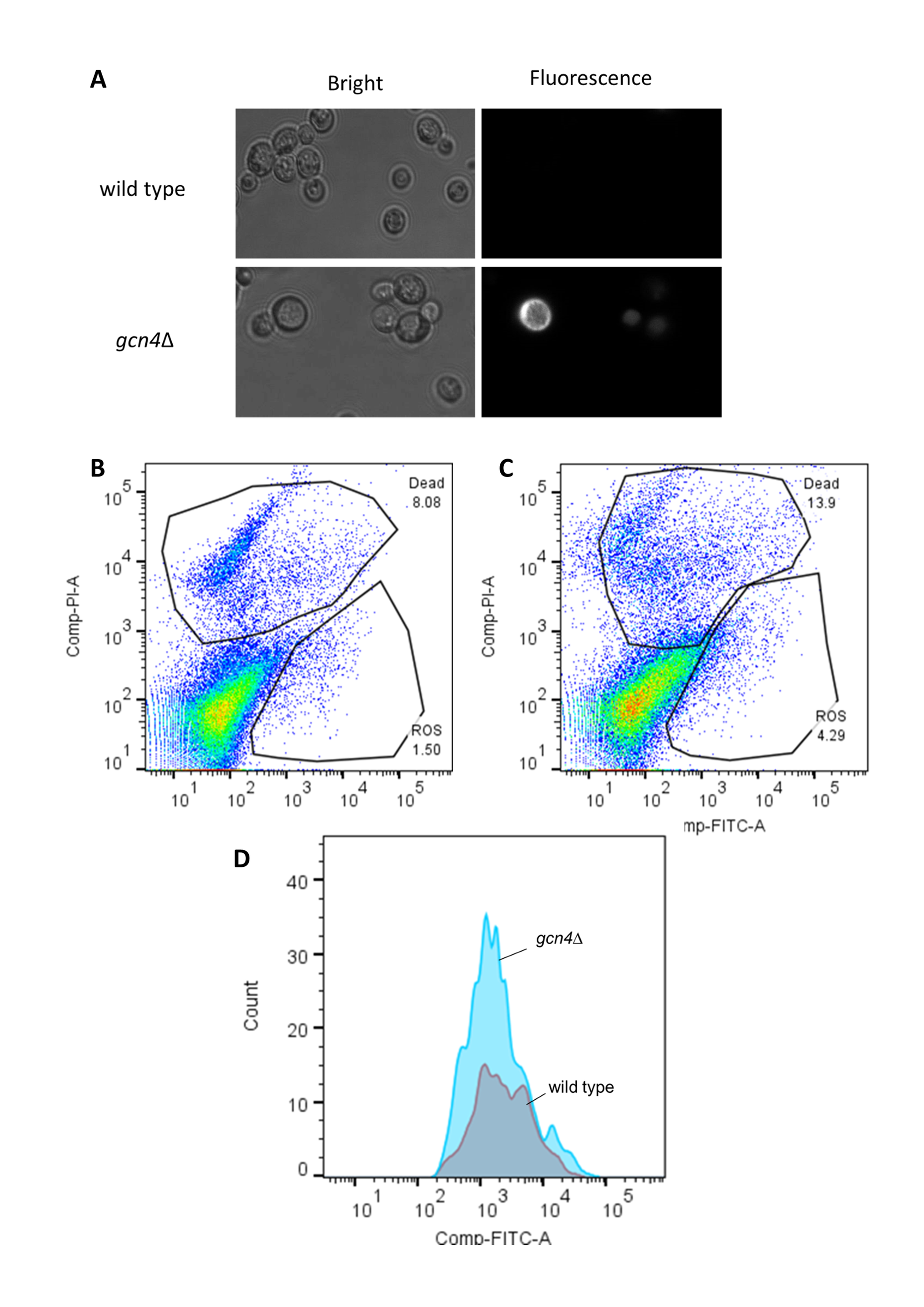

Supplement: S4 Fig — Representative images of cells grown in minimal medium without amino acids analyzed on a Zeiss LSM980 microscope equipped with a 63X (NA 1.4) and appropriate filter sets for acquisition of DCF (A). Representative graphs showing dot plots from flow cytometry analysis in wild-type (B) and gcn4∆ (C) cells grown in the presence of amino acids. Cells of interest were gated on SSC and FSC plot to remove any debris. ROS cells (positive for staining with H2DCFDA) and dead cells (positive for staining with propidium iodide) were defined by comparing with untreated cells. The x axis is a log scale of the intensity of H2DCFDA fluorescence and the y axis is a log scale of the intensity of propidium iodide fluorescence. The percentage of cells positive for each stain are indicated. In all cases, more than 10,000 cells were analyzed. D: Histogram comparing FITC signal of both strains. (TIF) [file pone.0292949.s004.tif]

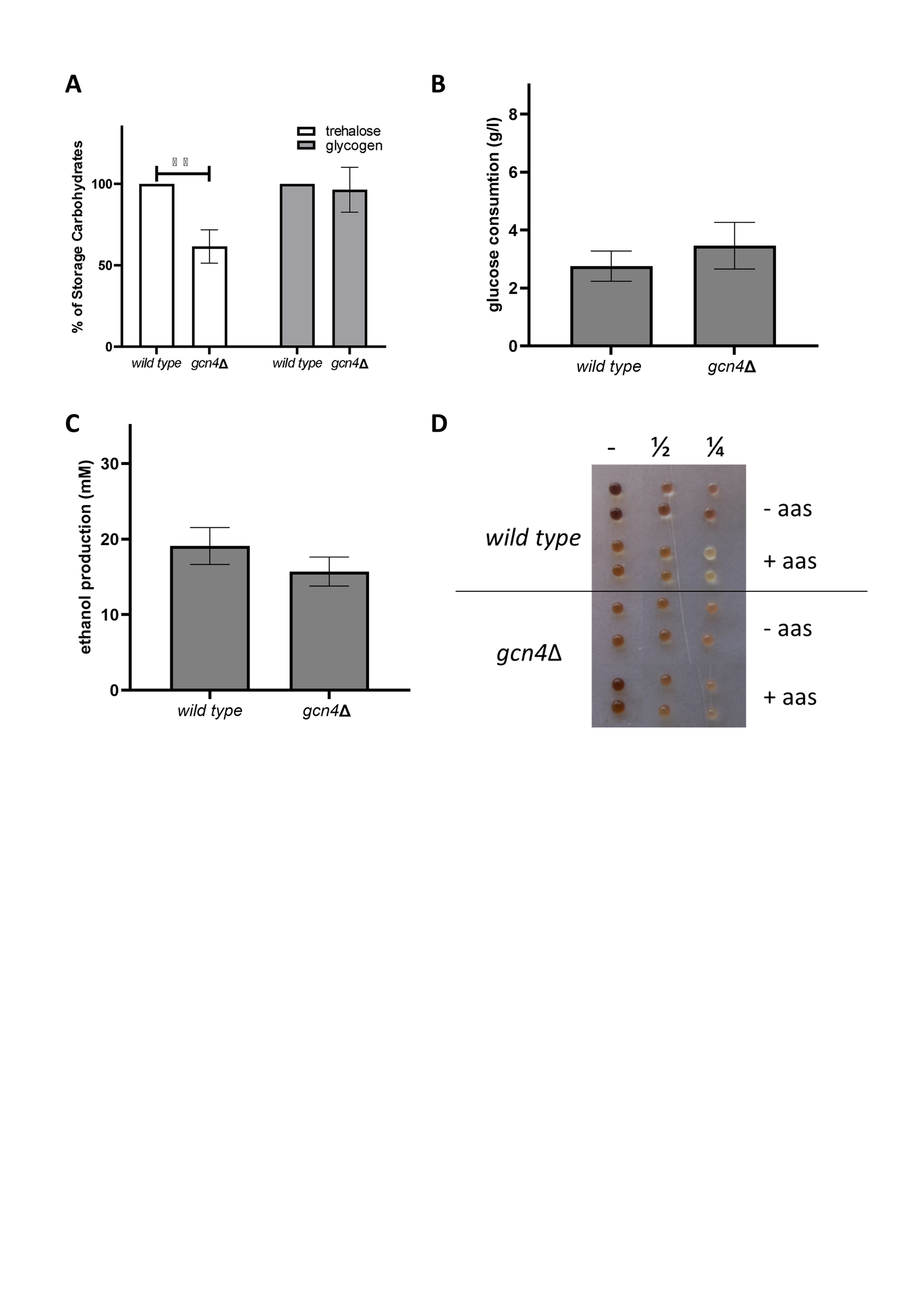

Supplement: S5 Fig — Cells were grown in minimal medium with amino acids A: Relative percentage of glycogen and trehalose contents in gcn4∆ cells with respect to wild-type cells at stationary phase (72 hours) grown in the presence of amino acids. B: Glucose concentration was determined in the culture medium supplemented with amino acids of wild-type and gcn4∆ cells at exponential growth phase (18 hours). Glucose consumption was calculated as the difference between the initial glucose concentration and the concentration in each tested condition. Values indicate the average of at least 3 independent experiments and the deviation indicates the standard error of the mean (SEM). The two-way ANOVA test was performed. C: Ethanol concentration (mM) was determined in the culture medium supplemented with amino acids of wild-type and gcn4∆ cells at exponential growth phase (18 hours). Values indicate the average of at least 3 independent experiments and the deviation indicates the standard error of the mean (SEM). The two-way ANOVA test (**: p<0.01) was performed. D: Representative image of one of the glycogen determinations. Samples were subjected to Lugol’s reagent. Duplicate seeding of wild-type and gcn4∆ cells treated with undiluted Lugol’s reagent, as well as half (1:2) and quarter (1:4) dilutions, are shown. (TIF) [file pone.0292949.s005.tif]
